# The Multifaceted Role of 3D Printed Conducting Polymers in Next-Generation Energy Devices: A Critical Perspective

**DOI:** 10.1021/jacsau.4c00796

**Published:** 2025-01-22

**Authors:** Nipun Jain, Yusuf Olatunji Waidi

**Affiliations:** † Department of Materials Engineering, Indian Institute of Science, C.V Raman Avenue, Bangalore 560012, India

**Keywords:** 3D Printing, conducting polymers, PEDOT:PSS, energy storage devices, biosensors

## Abstract

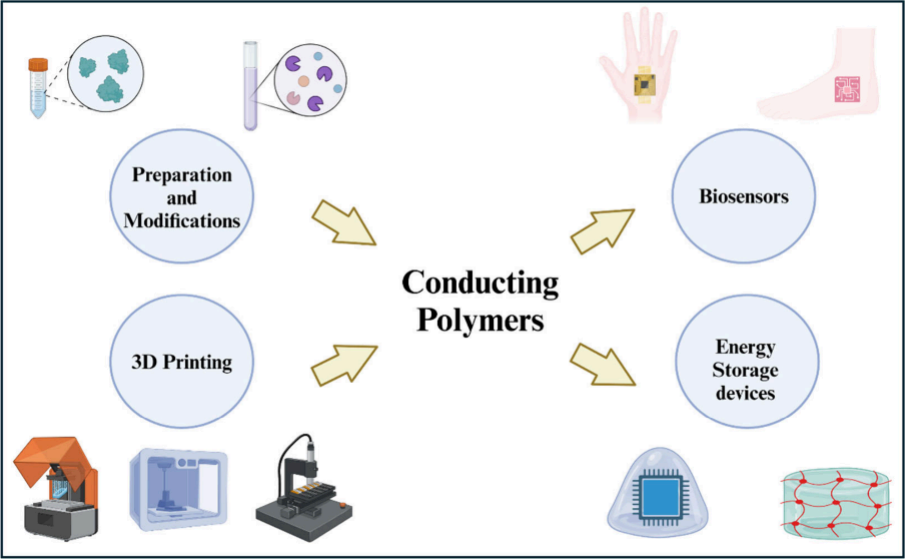

The increasing human population is leading to growing consumption of energy sources which requires development in energy devices. The modern iterations of these devices fail to offer sustainable and environmentally friendly answers since they require costly equipment and produce a lot of waste. Three-dimensional (3D) printing has spurred incredible innovation over the years in a variety of fields and is clearly an attractive option because technology can create unique geometric items quickly, cheaply, and with little waste. Conducting polymers (CPs) are a significant family of functional materials that have garnered interest in the research community because of their high conductivity, outstanding sustainability, and economic significance. They have an extensive number of applications involving supercapacitors, power sources, electrochromic gadgets, electrostatic components, conducting pastes, sensors, and biological devices thanks to their special physical and electrical attributes, ease of synthesis, and appropriate frameworks for functional attachment. The use of three-dimensional printing has become popular as an exact way to enhance prepared networks. Rapid technological advancements are reproducing patterns and building structures that enable automated deposition of polymers for intricate structures. Different composites have been created using oxides of metals and carbon to improve the efficiency of the CPs. Such composites have been actively investigated as exceptional energy producers for low-power electronic techniques, and by increasing the range of applications, they have verified increasing surface area, electronic conductivity, and remarkable electrochemical behavior. The hybridization with such materials has produced a range of equipment, such as gathering energy, sensors, protective gadgets, and storage facilities. A few possible uses for these CPs such as sensors and energy storage devices are discussed in this perspective. We also provide an overview of the key strategies for scientific and industrial applications with an eye on potential improvements for a sustainable future.

## 1 Introduction

The increasing demand for miniaturized and self-sufficient devices has spurred the development of alternative energy sources. Traditional batteries often fall short due to size, weight, and lifespan limitations. Consequently, researchers are exploring innovative energy solutions, including energy harvesting and storage technologies.^1−3^ Energy harvesting techniques, such as piezoelectric, thermoelectric, and biofuel cells, convert ambient energy into electrical power, extending device longevity and reduce reliance on battery replacements.^4−9^ Hybrid systems combining supercapacitors and biofuel cells offer promising, reliable, and self-charging power delivery solutions.^10,11^ Conducting polymers’ unique conductivity properties, biocompatibility, and flexibility are key materials in realizing these energy devices.^12−14^ Their incorporation enables the creation of conformable, efficient, and potentially implantable power sources for new generation of applications.

Sequel to the groundbreaking breakthrough of highly conductive doped polyacetylene in 1977,^15,16^ CPs have garnered significant attention from the scientific community, transitioning from laboratory curiosities to commercial realities. These materials comprise conjugated polymer chains with altered atomic structures to facilitate electrical conductivity. Prominent examples include poly(3,4-ethylenedioxythiophene):polystyrenesulfonate (PEDOT:PSS), polypyrrole (PPy), polyacetylene, and polyaniline (PANi). While inherently conductive, CPs often necessitate doping to achieve metal-like conductivity levels. To further augment electrical properties, conductive fillers such as carbon,^17^ graphene,^18−20^ and silver^21^ into CP matrices construct composites with tunable chemical and physical characteristics. The versatility of CPs, particularly PEDOT, PPy, and PANi, has driven their application in a wide array of energy devices and (bio)electronic, including biosensors, electrodes, electronic skin, human motion sensors, wearable technologies, soft robotics, and healthcare monitoring.

CPs possess unique physicochemical properties that offer unprecedented control over electronic device performance, making them promising candidates for next-generation electronics and sensors.^22^ These devices demand materials that can conform to body surfaces and accommodate movement without compromising electrical function.^23,24^ Traditionally, CP-based devices have been manufactured using solution-processing techniques like spin coating, drop casting, and aerosol printing.^25^ However, these methods are limited to low-resolution 2D patterns, often leading to topographical mismatches when applied to the skin.^26^ To bridge the gap between material processing and application, and potentially surpass inorganic materials in wearable and epidermal electronics, 3D printing has risen as a compelling alternative. This technique offers greater design freedom, enabling the fabrication of customized microscale structures such as honeycombs with improved processability and flexibility.

Additive manufacturing (AM), or 3D printing, has garnered enormous interest throughout various fields as a result of its ability to fabricate intricate structures with microscopic precision.^27,28^ By eliminating the need for traditional molds and dies, AM offers a direct digital pathway from design to product, streamlining the manufacturing process.^29^ Compared to subtractive lithography techniques predominantly used in electronics, AM allows the production of numerous constituents into a single unit, thereby reducing assembly complexities.^30^ Furthermore, AM’s potential to minimize waste and energy consumption compared to conventional techniques positions it as a sustainable alternative for electronic device production.^31^ Beyond electronics, the versatility and efficiency of AM have expanded its application spectrum to encompass industries such as food, energy, pharmaceuticals, chemicals, automotive, and aerospace.^32^

Recent literature has extensively explored the application of electrically conductive materials in energy devices. While some reviews have focused specifically on the role of electrically conductive polymers in additive manufacturing,^33,34^ others have encompassed both conductive polymers and composites.^35^

Nevertheless, their research did not fully investigate various module kinds and their particular uses in an enduring way. Furthermore, a thorough examination of the applications with the least amount of waste production is currently lacking. Therefore, in order to close this gap, the current Perspective offers a thorough examination at the intersection of conductive polymers, 3D printing, and manufacturing processes to address this gap. We highlight the unique properties of conductive polymers and goes on to discuss how these modules can be utilized in multiple sectors, such as biomedical and stretchable devices. In conclusion, the goal of this research is to present a thorough analysis of the developments, difficulties, possible uses, and prospects for 3D printing in these energy management systems. Additionally, we offer comparison reasons for improved comprehension and accessibility of 3D printing applications. We then provide an overview of the significance of this new class and point out future prospects. Lastly, we talk about how regenerative engineering might be revolutionized by incorporating electrical parts and sensors into flexible structures.

## 2 Fundamental Properties of Conducting Polymers

CPs constitute a class of organic materials offering a promising complementary to conventional conductive materials like metals and carbon-based compounds.^36,37^ Characterized by low density and excellent processability, CPs provide an efficient, conductive, and cost-effective base in multiple aresa, such as biomaterials, sensors, energy storage devices, and electronics.^38,39^ CPs can be categorized into intrinsic and non-intrinsic types, each further subdivided based on chemical structure and properties. Prominent families include polypyrroles, polyphenylenes, poly(arylene-vinylenes), polythiophenes, polycarbazoles, and polyanilines, each with distinct characteristics and applications. A subset of CPs, dual charge conducting polymers, facilitates ion and electron transport. The electrical conductivity of CPs arises from their conjugated structures, featuring alternating double bonds along the backbone.^40−42^ Three of the four valence electrons form localized σ bonds through sp2 hybridization, while the remaining electron occupies the pz orbital. Overlapping pz orbitals create π bonds, and the delocalization of π electrons along the conjugated system enables electrical conductivity akin to that observed in metals.^43^ Although intrinsically conductive, CP conductivity can be significantly enhanced through doping with p- or n-type dopants.^44^

**Figure 1:**
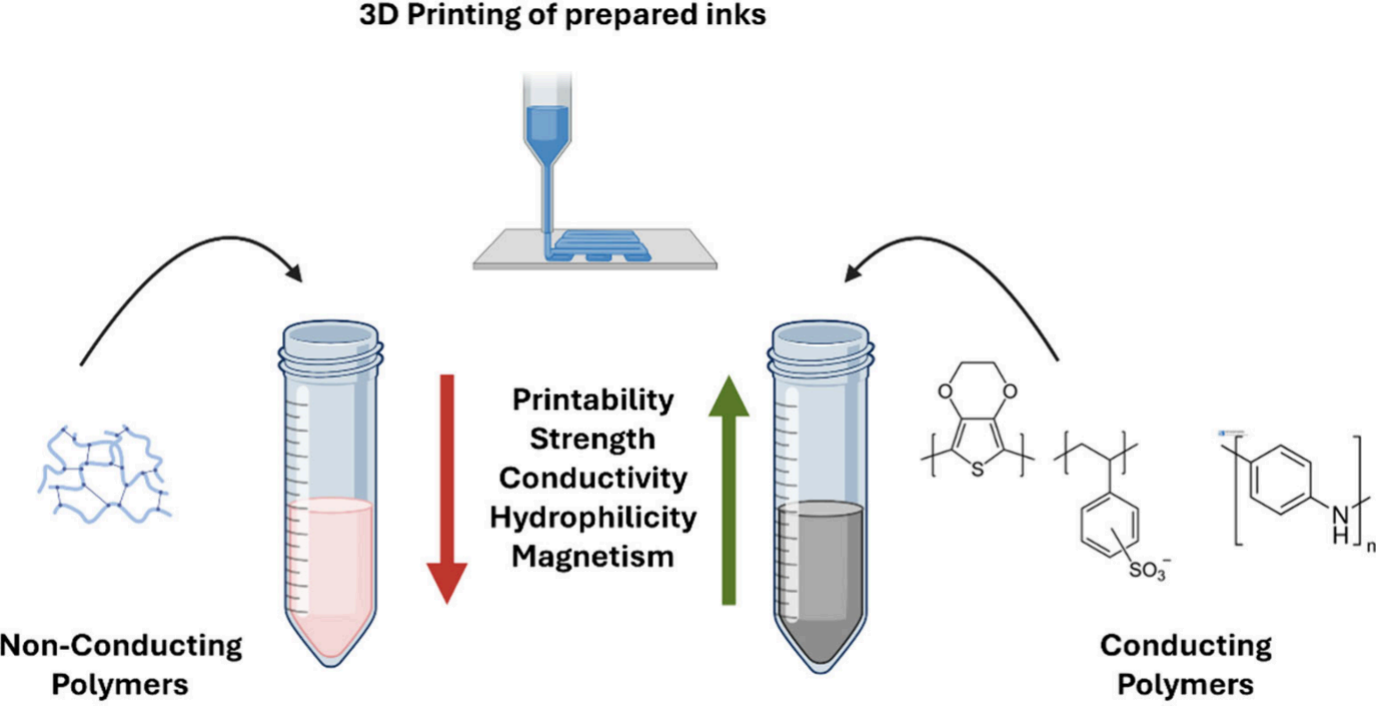
Schematic illustrating the influence of conducting polymers on 3D printing.

Intrinsic conducting polymers (ICPs) possess sufficient conductivity for many biomedical applications, but various factors significantly influence their electrical properties. Doping, especially with metal ions, enhances conductivity by strengthening the charge carrier concentration and modifying the polymer’s electronic structure.^37^ The polymer’s structural attributes, such as conjugated systems and crystallinity, also impact conductivity; for instance, chloroform-processed films often exhibit higher conductivity due to improved charge transport.^45^ Processing conditions, including solvent selection, temperature, and pressure, are crucial in determining polymer morphology and alignment, directly affecting charge carrier mobility.^46^ While temperature generally increases conductivity by promoting molecular motion, impurities can hinder charge transport, reducing conductivity.^47^ A comprehensive understanding and optimization of these factors are essential to develop high-performance conducting polymers for diverse applications (Figure 1).

ICPs offer a versatile platform for materials design due to their substantial structural diversity.^48,49^ The polymer backbone’s length, linearity, and branching significantly influence conductivity and mechanical properties. For example, terpolymers incorporating noncentrosymmetric units have demonstrated enhanced ductility and charge mobility, even under strain.^50^ This structural tunability and the ability to modify chemical composition through functionalization enables precise tailoring of electrical, optical, and chemical properties..

Polyaniline (PANI) is a promising intrinsically ICP as a result of its low-cost production, facile synthesis, environmental stability, and tunable properties.^51,52^ These attributes have spurred interest in its integration into additive manufacturing processes as a primary material and filler. Despite its versatility, PANI’s insolubility and infusibility, common challenges for π-conjugated polymers, hinder its processability and limit commercial applications.^53^ To address these issues, modifications such as incorporating polar groups into the polymer backbone can enhance solubility and processability. Polypyrrole (PPy) is another extensively studied ICP known for its high conductivity, facile synthesis, environmental stability, and biocompatibility.^54^ However, its π-conjugated structure leads to similar processability challenges as PANI. Strategies to improve PPy’s processability include monomer modification, copolymerization, and doping.^55^ While these approaches can enhance solubility, the introduction of large dopants to reduce intermolecular interactions often compromises electrical conductivity by elevating the distance between charge carriers.^56,57^ Poly(3,4-ethylenedioxythiophene) poly(styrenesulfonate) (PEDOT:PSS), a derivative of the π-conjugated polythiophene, has emerged as a leading ICP due to its exceptional chemical and thermal stability, corrosion resistance, low cost, biocompatibility, transparency, conductivity, and flexibility.^58^ PEDOT is inherently insoluble and synthesized via chemical or electrochemical oxidation of EDOT.^59^ The addition of PSS imparts water solubility, enabling solution-based processing.^60^

In a nutshell, ICPs offer several advantages over conventional conducting materials for biomedical energy devices (Table 1). Their biocompatibility is particularly noteworthy, as many ICPs demonstrate excellent compatibility with biological tissues, thereby minimizing adverse implant reactions. Moreover, the processability of ICPs is exceptional, enabling the fabrication of diverse forms such as films and fibers, a crucial aspect of device design and development. While exhibiting lower intrinsic conductivity compared to metals, ICPs can attain sufficient conductivity through doping, rendering them suitable for a variety of purposes. The inherent flexibility of these polymers allows for the creation of conformable devices that seamlessly integrate with the body, enhancing both comfort and functionality.^61^ Additionally, ICPs are lightweight and often more cost-effective than traditional materials, making them attractive options for implantable devices. The combination of these properties establishes ICPs as promising materials for advancing biomedical energy technologies.

**Table 1:** Properties of Mainstream Conductive Polymers

Property	PEDOT:PSS	Polyaniline (PANI)	Polypyrrole (PPy)
Conductivity	Moderate to high conductivity, especially when doped with PSS (1–1000 S/cm)^62^	Can exhibit high conductivity, but depends on doping level and oxidation state (1–1000 S/cm)^63,64^	High conductivity, but can be sensitive to environmental factors (10–1000 S/cm)^65,66^
Solubility	Soluble in water and some organic solvents (due to PSS)^67,68^	Poorly soluble in most solvents, often requires specific doping agents or chemical modifications^69−72^	Generally insoluble but can be dispersed in certain solvents or processed electrochemically^73−75^
Doping Method	Chemical doping with PSS during synthesis^76^	Chemical or electrochemical doping with acids (e.g., HCl, H_2_SO_4_)^63,77−79^	Chemical or electrochemical doping with anions (e.g., Cl^–^, BF_4_^–^)^80−82^
Stability	It has good environmental stability but can degrade under prolonged exposure to light or heat.^83,84^	Can degrade under exposure to light, heat, and certain chemicals^85,86^	Can be sensitive to environmental factors, particularly humidity and oxygen^87−89^
Optical Properties	Transparent to near-infrared^90−92^	Color-changing (blue to green)^93,94^	Color-changing (dark blue to black)^95,96^
Robustness	Flexible and mechanically robust^97,98^	Flexible and mechanically robust^99^	Flexible and mechanically robust^100^
Processability	Easily processable from aqueous solutions^101^	Often processed from organic solvents or water-based dispersions^102,103^	Often processed electrochemically or from organic solvents^75,104^
Biocompatibility	Generally considered biocompatible but can depend on specific applications and formulations.^105−107^	Biocompatible but can be cytotoxic at high concentrations or with certain dopants^108,109^	Generally biocompatible, but can induce inflammation or allergic reactions in some cases^110,111^

## 3 Bioink Requirements and Different Printing Techniques

The successful implementation of 3D printing heavily relies on the formulation of well-designed inks. Key factors such as rheological properties, chemical compatibility, solidification mechanisms, material properties, print resolution, and shelf life significantly influence the printing process and the final product’s quality. Inks must have appropriate viscosity to ensure smooth extrusion and maintain postdeposition shape.^112^ Shear-thinning behavior is crucial, as it allows the ink to decrease viscosity under shear stress, facilitating the extrusion process.^113^ Chemical compatibility with printer components and additives is essential to prevent clogging and maintain the integrity of the printing process.^114,115^ Solidification mechanisms, such as thermal or UV curing, are necessary to transform the liquid ink into a solid structure postdeposition.^116^ In some cases, additional postprocessing steps may be required to enhance the final product’s properties. The material properties of the printed objects, including mechanical strength, thermal stability, and chemical resistance, are critical for various applications. To achieve high-resolution prints, the ink’s particle size and the printer’s nozzle size must be carefully matched. Additionally, inks must exhibit long shelf life and storage stability to maintain printability. While these requirements are essential for successful 3D printing, challenges persist in optimizing ink formulations for specific applications, particularly in the biomedical field, where bioactivity and compatibility with living tissues are paramount.

### 3.1 Extrusion-Based Printing

This technique has been proposed to be the best technique for producing thin filament structures. It has proven to be very useful as it provides an extensive range of components that can be easily printed.^117^ Polymer-based inks are accurately deposited onto the surface using ejection forces. In this way, filaments are continuously produced that may be precisely layered.^118^ It can apply several inks to a single structure, enabling various combinations crucial for simulating the intricate structure. Additionally, the scalability allows for the production of constructions required for practical substitutes. Considering these advantages, several uses have been documented in biomedical engineering, education, and prototype development, suggesting further promise with the advancement of related facilities.^119^

### 3.2 Droplet-Based Printing

In this technique, depositing position and droplet size can be controlled by using temperature or piezo-inkjet controllers. The droplet is created and deposited by overcoming the surface tension. The volume, impact velocity, and polymer characteristics are additional variables that affect printing.^120^ Low-viscosity inks can occasionally produce fragmented drops that call for preprinting cross-linking techniques. An additional challenge is the requirement to create strategies for combining distinct units. On the contrary, the tiny nozzle restricts focus and production time, which raises the possibility of blockage inside the nozzle and necessitates periodic cleaning. Droplet-based printing has limited prospects in light of these variables, such as in biomedicine, electronics, and material science.^121^

### 3.3 Laser-Based Printing

Using this method, high-pressure droplets are produced on a laser-energy-absorbing layer by a beam of a specific wavelength. The generated droplets form a jet motion and are propelled toward a collecting substrate, through which highly accurate patterns can be produced.^122^ A concentrated laser beam is transmitted from a donor strip to a receiving surface. The main advantage is its precision and accuracy when building high-order intricate designs. However, large-scale manufacturing is hampered by low scalability and expensive cost.^123^ These difficulties result from the requirement for exact system controls and advanced laser networks, which can be costly and difficult to operate. Furthermore, metal deposits may be present in uncommon patterns, significantly raising the scale-up factor. Therefore, in order to improve output, these issues should be fixed.

### 3.4 Light-Based Printing

This technique uses UV or visible light to polymerize and preferentially cure photosensitive materials using computer-designed models.^124^ The selection of photoinitiators is important since it affects the efficiency of the treatment. It needs to be precisely adjusted as a high concentration can produce too many free radicals, and a low concentration can result in inadequate cross-linking.^125^ Overall, the benefits include its high accuracy, and adaptability, which enable the careful rebuilding of intricate 3D models. It is also important to consider the possible degradative effects of UV light, and it becomes necessary to address the restricted choice of photopolymerization-responsive inks.^126^ It can create a fresh path for development by attaining flexibility for broader programs.

Many printing processes have shown promising results, industrial use, and economic viability because of their great adaptability, scalability, and affordability. They occasionally have limitations when it comes to extensive rebuilding. When combined, printing methods can be investigated concurrently to overcome challenges, which is crucial for investigating different polymers.

## 4 Applications of Conducting Polymers in Sustainable Energy Devices

Multipurpose conducting polymers are utilized in multiple programs, including soft robotics, regenerative medicine, bioelectronic sensors, and energy storage devices. Various recent studies and their concluding remarks have been compiled in Table 2. The advances in this area are covered below:

**Table 2:** Interesting Studies on Conducting Polymers for Biomedical Energy Devices

Materials	Printing Technique	Application	Remark	Ref
PEDOT	Extrusion	bioenergy harvesters, biosensors, and biomedical devices	The immobilized bacteria within an electropolymerized PEDOT matrix amplify bioelectrochemical responses. 3D printing significantly decreased electrode fabrication time from several hours to 35 min. Cyclic voltammetry measurements demonstrated enhanced current generation by the living electrode compared to a control.	(127)
PLA, PEDOT, and poly(hydroxymethyl-3-ethylenedioxythiophene) (PHMeDOT)	Extrusion	electrochemical sensors	The fabricated PLA/CP-Aq sensor exhibited excellent electrochemical performance in detecting dopamine (DA) using various systems, such as differential pulse voltammetry (DPV), chronoamperometry (CA), and cyclic voltammetry (CV). The sensor demonstrated selectivity toward DA in the presence of interfering ascorbic acid (AA) and uric acid (UA) species.	(128)
PEDOT:PSS, Polyacrylamide (PAAm), and PHEA coating	digital light processing (DLP)	electroluminescent devices and capacitive sensors	Integrating a PEDOT: PSS network within the PAAm gel confers superior electrical conductivity, while a PHEA coating safeguards this conductive layer from short circuits and physical harm. The hybrid material demonstrates exceptional stretchability, minimal hysteresis, and robust adhesion between the PHEA components and hydrogel.	(129)
PEDOT:PSS	Extrusion	illuminating wristbands, location trackers, and wearable biomedical sensors	A novel, rapid method produces high-performance 3D microsupercapacitors (MSCs) in just 6-h, significantly outperforming conventional 72-h processes. The developed optimized, highly conductive hydrogel enables the creation of MSCs with superior energy density, scalability, and reproducibility.	(130)
PEDOT:PSS/PEGDA and indium tin oxide-coated polyethylene terephthalate (PET-ITO)	stereolithography (SLA)	wearable biomedical sensors	The developed sensor demonstrated a linear, highly sensitive, ultrafast response and biomedical application potential. Its ability to detect minor pressure differences (as low as 1.5 kPa), including those generated by electrically induced contractions in a bioactuator, confirms its suitability for precise pressure sensing.	(131)
PEDOT:PSS, PVA, and dopants poly(sulfobetaine methacrylate) (PSBMA)	Extrusion	soft bioelectronics	The fabricated device exhibited exceptional properties, including high conductivity (1.2 S m^–1^), low interfacial impedance (20 Ω), outstanding stretchability (349%), and superior toughness (109 kJ m^–3^). It also demonstrated strong adhesion to diverse materials and significantly outperformed commercial alternatives in high-fidelity recording ECG and EMG signals.	(132)
PEDOT:PSS, poly(vinyl alcohol-formaldehyde) (PVAF)	Extrusion	soft bioelectronics	The device demonstrated superior adhesion (31.44 ± 7.07 kPa), conductivity (>100 S m^–1^), and electrochemical properties (13.72/18.80 mC cm^–2^ charge injection/storage). When fabricated into adhesive skin electrodes, it outperformed commercial counterparts in electromyography (EMG) signal recording, consistently providing high signal-to-noise ratios (>10 dB) across various conditions.	(133)

### 4.1 Bioelectronics and Tissue Engineering

**Figure 2:**
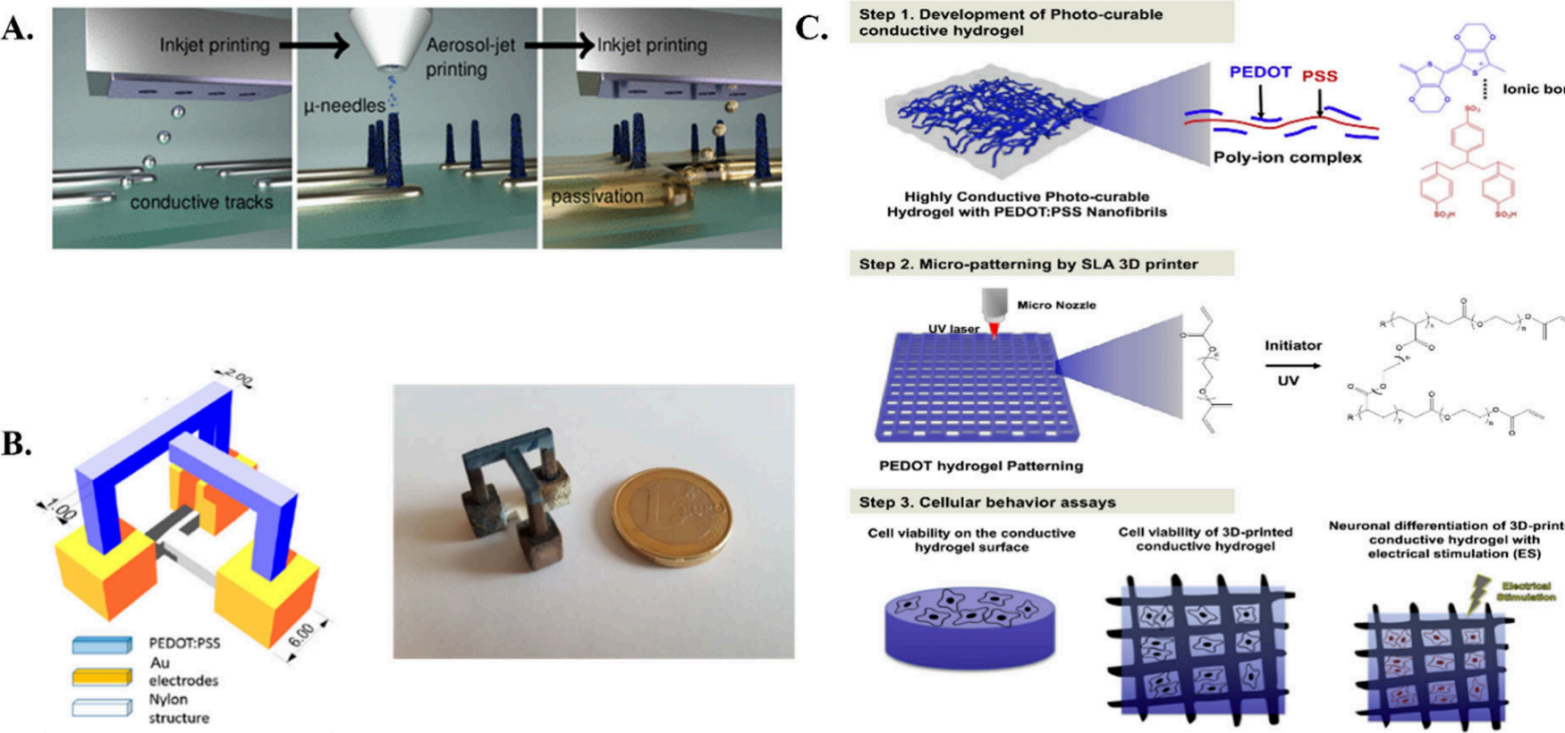
A. Schematic representation of 3D inkjet and aerosol printed micro electrode array for bioelectronic applications. Reproduced with permission from ref (139). Copyright 2019, American Chemical Society. B. Schematic and the optical image of the 3D gadget that was built and manufactured. Reproduced from ref (136) under CC BY 4.0. C. Diagram showing the steps involved in creating a 3D conducting device utilizing cellular behavior tests and a SLA printing equipment. Reproduced with permission from ref (138). Copyright 2019, Elsevier.

CPs have risen as novel substances for applications in supercapacitors, sensors, stretchy transistors, and electronic skins. Because CPs lack the natural flexibility and stretchability required, they are frequently combined with another polymer—silicon or elastomer—to improve their mechanical characteristics while preserving electrical conductivity.^35^ There have been numerous reports in the scientific literature on using conductive polymers. Because of their great sensitivities, cheap manufacturing expenses, and capacity to detect significant concentrations of volatile chemicals, CPs can be used as sensor layers.^134^ Recently, studies have been done on the 3D printing of digital airflow sensors using CPs. PEDOT: PSS-based hair-like elements were 3D printed in order to mimic the airflow sensors found in nature.^135^ In reaction to airflow, a collection of these sensors functioned as valves to a central terminal. Tarabella et al. provided another instance of how PEDOT: PSS can be used for resistor humidity detectors. An SLS-manufactured composition is prepared and later coated with PEDOT:PSS^136^ (Figure 2B).

Multiple studies on AM CPs have implications in the medical area, such as orthopedic implantation sensors.^137^ Tissue engineering is an important field in which conducting polymers have been 3D printed using techniques like SLA (Figure 2C). It often leads to a decrease in cell toxicity, as evidenced by a rise in cell growth, which exhibits no discernible harmful effects.^138^ Using a different strategy, a similar composite was reported that was additively synthesized for bioelectronics applications. The electrode tips (μ-needles) were created by combining inkjet and aerosol printing to detect electrophysiological recordings^139^ (Figure 2A).

Perforated guide conduits were made from a conductive composite of PPy and polycaprolactone to repair peripheral nerve injury using an electrohydrodynamic-jetting printer.^140^ The mechanical, electrical, and toxicity characteristics of the printed structures for nerve growth were investigated. It was demonstrated that neural crest stem cells adhere and develop into peripheral neurons more efficiently. Ma and coauthors investigated the impact of polypyrrole particulate concentration.^141^ Particles of different morphologies were dispersed in a PLLA scaffold and printed using direct ink writing. In contrast to spherical morphology, they discovered that tubular ones had higher conductivity, which might be useful in related applications. Similarly, conducting polymers with an inkjet-printed PPy/collagen composite was studied.^142^

### 4.2 Biosensors and Electrophysiology

**Figure 3:**
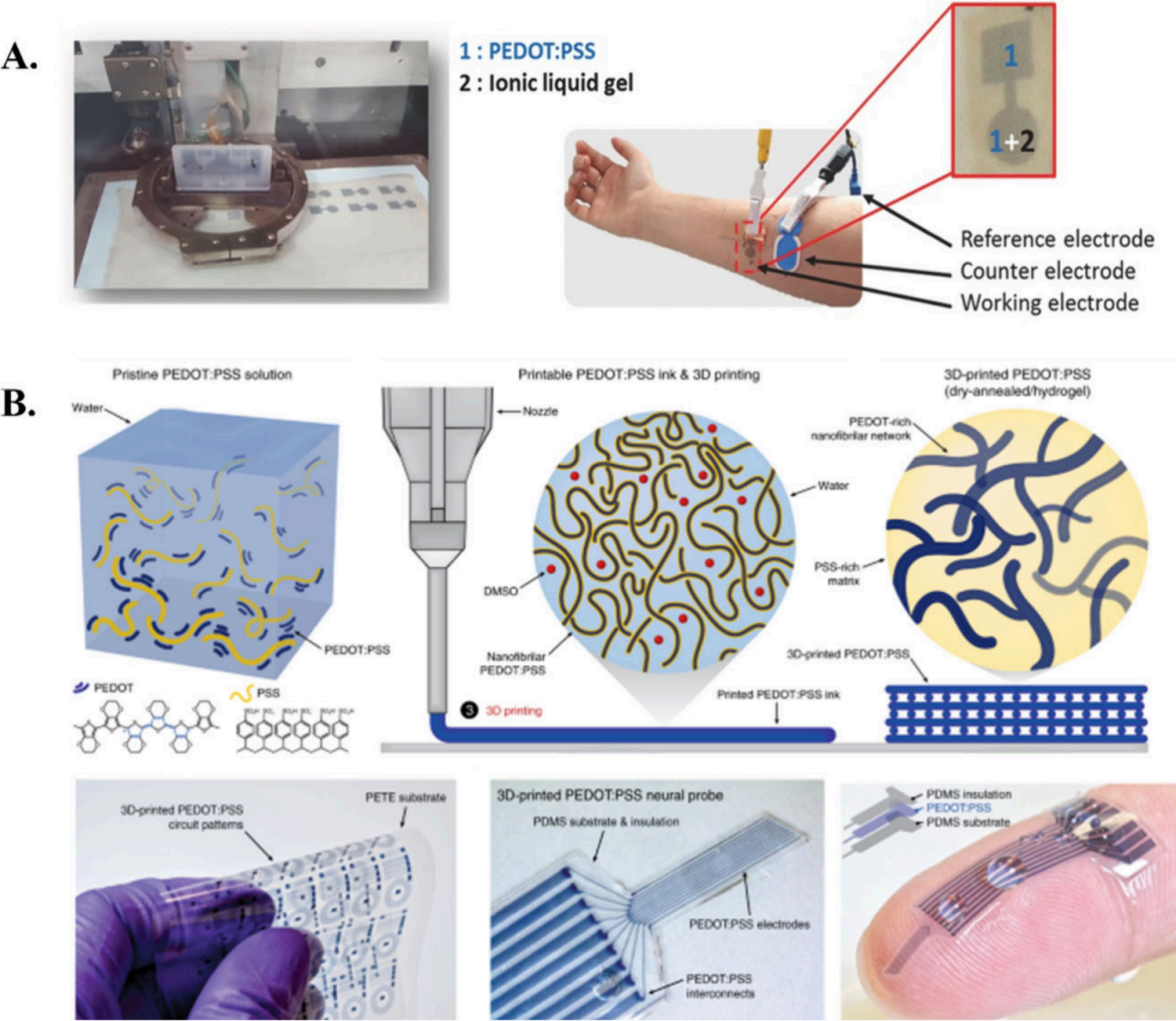
A. Inkjet printing procedure and displaying the electrode arrangement used for the impedance tests. Reproduced from ref (143) under CC BY 4.0. B. Pristine PEDOT:PSS solution is shown to be 3D-printed both in dry and moist conditions. Folding of the printed circuit without failure is also possible. Reproduced from ref (144) under CC BY 4.0.

The extensible and stretchable electronics sector is expanding to create wearable technology that is lightweight and resilient to high-stress levels. For instance, a PEDOT: PSS-based device that records physiological information was manufactured using inkjet printing (Figure 3A). Electrocardiograms (ECGs) were recorded in order to evaluate this wearable technology.^143^ Additionally, an ionic gel ink was selected and imprinted on the upper layer to increase the interaction among conduction polymer and the skin. This type of gel produces superior linkages that are stable over time. Overall, the distinct benefit of not being dehydrated and using an arrangement more suited to wearable evaluations is that these gel-obliged electrodes produced readings with performance similar to commercial Ag/AgCl electrodes while making low-impedance connections to the skin. These findings enable the application of the intended electrodes as adaptable health detection tools for electrophysiological data to be validated.^143^ PEDOT:PSS microscopic structures printed by 3D printing can also be used as a neuronal setup for in vivo recordings. In this instance, a surgically implanted sensor was used to study electrophysical phenomena (Figure 3B). The 3D-printed neural device was connected to Neuro Nano Strip Connectors for electrical monitoring. The findings demonstrated its ability to continuously capture neural activity in a mobile mouse.^144^

The 3D printing of PPy using DLP was studied for possible applications in sensing.^145^ In this case, urethane dimethyl acrylate (UDMA) was mixed with PPy to enhance the stiffness. Although it hampered overall conductivity, the UDMA also helped with 3D printing. The authors recommended that this method be appropriate for sensor-related applications in the future. A 3D-printed PANI structure was also studied for possible sensing applications.^146^ The DIW approach was used to print PANI, which was filled with DBSA and treated with thermal doping to improve conductivity.

### 4.3 Electrochemical/Energy Storage Devices

**Figure 4:**
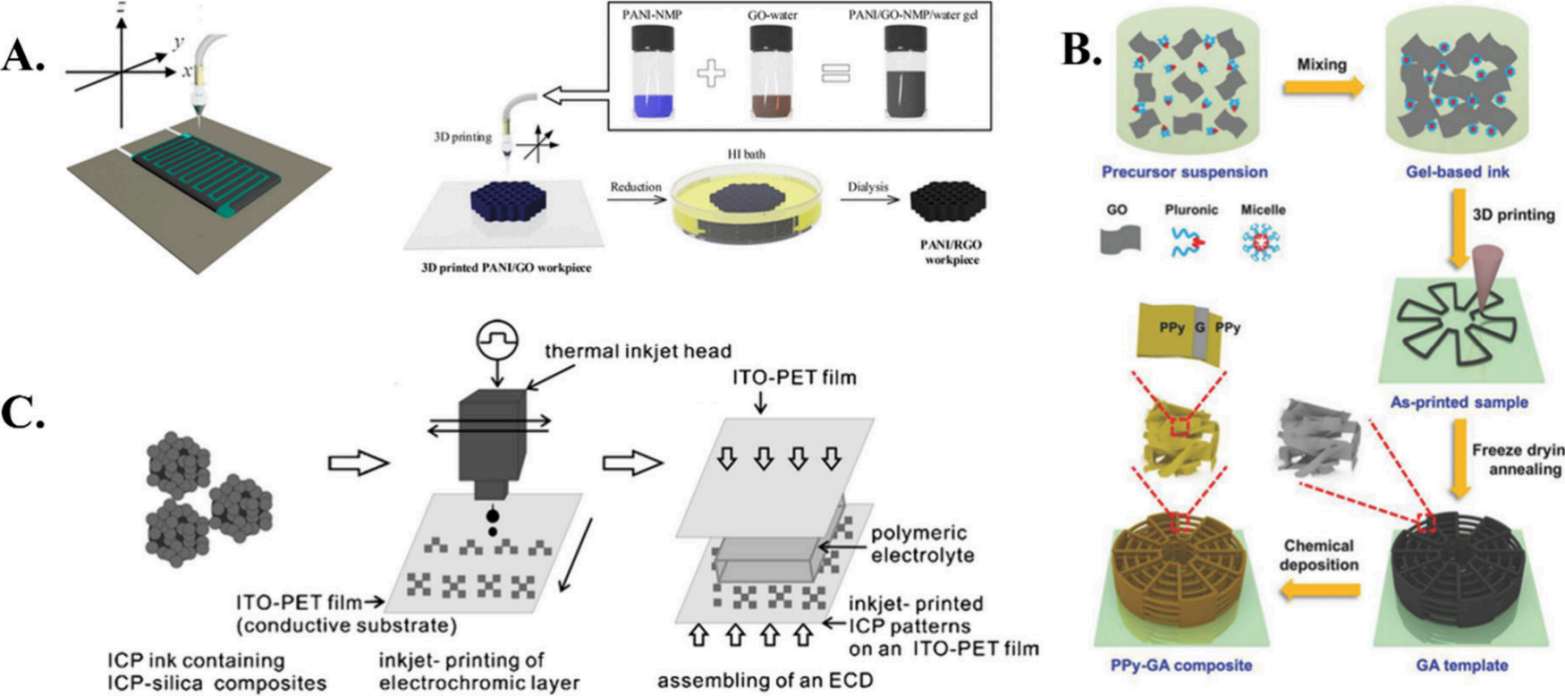
A. 3D printed constructs of PANI and RGO composites in which the printable inks are built by self-assembly. Reproduced with permission from ref (148). Copyright 2018, American Chemical Society. B. Diagrammatic illustration of the three-dimensional PPy–GA composites’ manufacturing method. The printed construct underwent gelation, freeze-drying, and thermal reduction to produce a convertible GA design. Reproduced from ref (149) under CC BY 4.0. C. Schematic representation process for creating an electrochromic layer (ECD) by inkjet printing. Reproduced with permission from ref (151). Copyright 2008, Royal Society of Chemistry.

The production of these devices by additive manufacturing presents the distinct benefit of intricate layouts with large surface contact. The conducting polymers reported have much promise because of their excellent electrode interactions between the ionic components. These hybrid substances can be utilized to preserve the performance of energy-storing devices while enhancing the general processabilities in 3D printing.^147^ Graphene oxide and PANI hybrid potential were shown in the 3D printing of energy-storing devices. A 3D adaptive matrix was formed in this work using graphene oxide sheets (Figure 4A), where strong π–π interactions drove PANI chains to assemble precisely on the formed sheets.^148^ These findings suggested possible energy device and supercapacitor uses.

For electrochemical energy storage, Qi et al. similarly employed direct ink writing to generate PPy-graphene-based devices (Figure 4B). Nevertheless, PPy was employed in this study to coat the printed graphene–aerogel framework instead of distributing it directly. Although the huge surface area of the multilayer spongy structure was stated to be beneficial, the coating of the active ingredient PPy was primarily concentrated on the outermost layer instead of the tiny inside gaps.^149^ Additionally, improved compression and complete restoration of the printed products were made possible by the permeable, lightweight complex. This innovative method may be used in fuel cells, solar cells, and batteries. In contrast, Lu et al. used selective laser melting (SLM) to create a supercapacitor for porous iron–nickel/polyaniline nanocages. Here, 3D printing was used to create a permeable topology for the Ni alloy, which was subsequently coated with PANI using electrochemical polymerization.^150^ A PANI/silica and PEDOT/silica composite-based inkjet printed electrochromic device was studied. The devices’ active color-changing surface was printed using an inkjet printer because of the solvent-free colloidal suspension of the CP. When a voltage was supplied to the printed device, the color changed from −2 to +2 V for both mixtures (Figure 4C). When the various voltages were applied, the observed color shift matched the CPs’ redox condition.^151^

Lithium ion-based batteries were also made using direct ink writing of PEDOT:PSS and CMC-based electrodes. With 100 cycles, this electrode keeps 92% of its capacitance, demonstrating a high durability and high areal potential of 5.63 mAh cm–2. According to Cui and colleagues, charge transfer and electrolyte infiltration are facilitated by the tortuosity or square pore geometry, which offers efficient transport pathways for ions.^152^ Solar cells have been made using electrodes produced through the 3D printing of conducting polymers. In this instance, Ag-NWs are positioned at both sides of the PEDOT:PSS and printed using an inkjet printer. This leads to the development of solar cell technology with high-power transformation.^153^ In another case, organic photovoltaic cells with a high surface area of 186 cm2 were created using the previously described strategy, with Ag positioned at both sides. These cells also demonstrated power transformation of 1.6% and excellent reliability even at low sunlight.^154^

### 4.4 Wearable Energy Storage Devices

**Figure 5:**
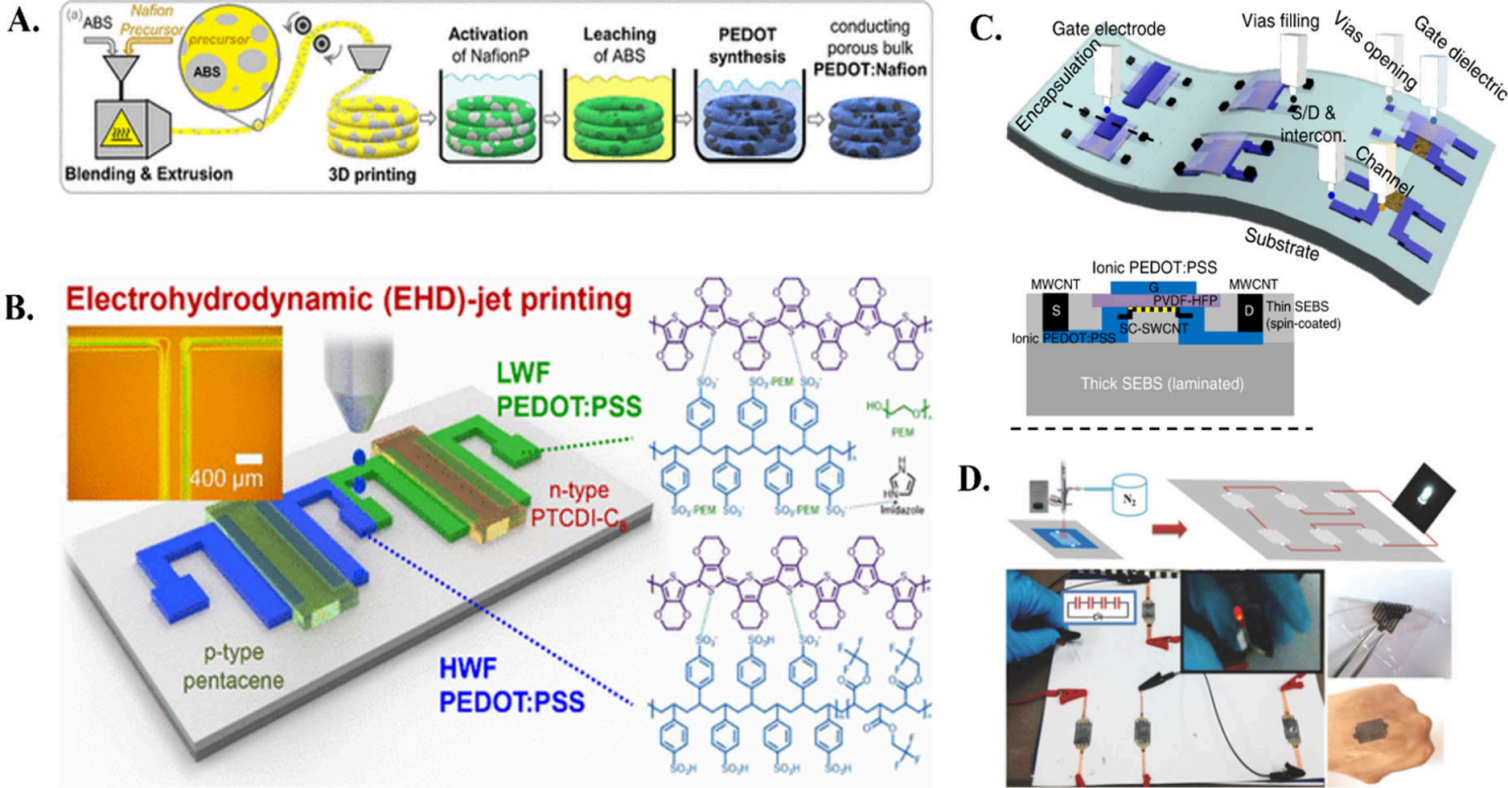
A. The flow of process for the fused filament fabrication of the precursor/ABS mixture to produce printed constructs of PEDOT:Nafion. Reproduced from ref (155) under CC BY 4.0. B. Diagram showing the steps involved in creating a supplementary NOT gate using PEDOT:PSS conductors with various optical microscope pictures of the electrodes printed using EHD jet on Si/SiO2 surface. Reproduced with permission from ref (156). Copyright 2020, American Chemical Society. C. The gadget’s cross-sectional schematic shows the labeling of every single layer and the overall idea and layout of the stretchable matrix of semiconductors shows how each layer was 3D printed using a similar technique. Reproduced from ref (157) under CC BY 4.0. D. A new method for printing graphene and CP hybrids inks to create in a plane MSCs. Reproduced with permission from ref (159). Copyright 2016, John Wiley and Sons.

3D printed CPs are additionally used in various domains like photodetectors, supercapacitors, transistors, wearable storage devices, and more. A major consideration is for the polymer to function without failing under severe strain. CPs are frequently mixed with another copolymer to improve their physical characteristics while preserving conductivity because they are not naturally flexible to the degree required for electronic purposes. Using PEDOT 3D printing, organic electrochemical transistors (OECTs) can be produced (Figure 5A). The conductivity (3 S cm^–1^) of Nafion fibers was retained even after stretching them to 100% elongation.^155^ In a different instance, the EHD of PEDOT:PSS produced OECTs with exceptional electrical characteristics, such as high carrier mobility and a switching ratio greater than 107 (Figure 5B).^156^

A different illustration centers on producing flexible PVDF, PEDOT:PSS, and SWCNT transistors (Figure 5C). These transistors showed high mobility at low operating voltage.^157^ In addition, a similar hybrid of PEDOT:PSS (healing agent) and DMSO (electric enhancer) were 3D printed to create elastic and regenerating wearable thermal electricity generators (TEGs).^158^ Moreover, graphene-mixed PEDOT:PSS has shown high capacitance and can be used as an microsupercapacitors (MSC) (Figure 5D).^159^ The direct ink writing of PPy results in supercapacitors with excellent execution in a high area capacitance of up to 93% after bending.^160^ The addition of graphene oxide in the PANi has been used to create adjustable MSCs achieving high capacitance.^161^

According to a recent study, microreactive inkjet printing can be used to 3D print PEDOT: PSS/ionic liquid hydrogel composite for flexible and wearable electronics.^162^ PEDOT: PSS composite can also be imprinted onto an elastic, which may be an application in flexible electronics. The printed structures can shrink to their initial shape after withstanding expansions of up to three millimeters before breaking. Conductive polymer penetration onto the printed structures could also create CPs. Hoffman et al. showed this by combining ABS and a 3D printed Nafion template using FFF, and then immersing the mixed portion in a PEDOT bath.^155^ By using this method, conductive PEDOT was able to fuse with the Nafion framework and reach a conductivity of 3 S cm–1, enabling the creation of conductive components for wearable electronics, e-textiles, and organic electrochemical transistor (OECT) devices.

## 5 Feasibility for Next Generation and the Road to a Sustainable Future

While 3D printed composite materials have tremendous potential for many uses, there are concerns about sustainability, safety, and effective translation on their path to becoming widely used sensors and energy storage devices. Issues about sustainability are raised by the substances and manufacturing techniques used. 3D printing is a highly environmentally friendly process through its capacity to reduce waste production, energy consumption, and greenhouse gas emissions.^163^ It also makes it possible to create equipment and their individual parts with the least amount of solvents by reprocessing the same components for future manufacturing phases. Crucially, 3D printing leverages the notion of layer fabrication instead of slicing things from bigger portions, as is typically done by traditional methods, which results in less wastage. Because the technology behind 3D printing is AM, it does not produce scraps or material loss as a result of faults.^164^ The technique’s sustainability is also influenced by the substances employed for manufacturing. Natural polymers are continuously being employed to achieve a more environmentally friendly manufacturing process. Additionally, 3D printing is a quick and straightforward procedure that uses less energy and emits less CO_2_. A revolutionary 3D printer called Eco Printing is also under development, employing discarded polymers as printing ink and utilizing a solar-charged device for fuel with the goal to lower greenhouse gases.^165^ Having the capacity to execute on-site printing also lowers greenhouse gas emissions during transportation. Given its ability to produce goods with little waste and no need for additional compounds, it is evident from the aforementioned instances that this technique would take a significant step toward a growing economy.

Although 3D printing and other manufacturing methods provide fine control, their usage of chemicals and substantial costs make them unsuitable for mass manufacturing. Although the preparation of energy devices is currently in its early stages, one of the key areas that need to be investigated is their adaptability for real-world applications. Nevertheless, a variety of constraints, including production costs, the speed of the approach, challenges with commercialization, and the need to keep the same functionality at an extensive level, may hinder 3D printing’s potential.^166^ For example, the expenses associated with purchasing printers and the intricate postprocessing procedures, particularly when using polymer-based approaches, can raise the cost of manufacturing tasks. Manufacturing flatter, bigger prints are a significant hurdle as current technology have a narrow printing height.^167^ Investigating various inks and optimizing the printing conditions for sustainable operations are therefore crucial. Furthermore, due to their time-varying modifications, these complex structures require a thorough assessment of any unexpected reactions. One persistent challenge is increasing output while preserving complex design elements and functionality. Moreover, techniques to improve the resilience of certain structures are required for prolonged use due to their intrinsic instability, which makes them susceptible to swelling-induced structural collapse or early deterioration.^168^

Furthermore, the program utilized by 3D printers is difficult to build up for everyday use because it demands a high degree of knowledge. In order to overcome these obstacles, the combination of outside integration systems with large-scale, sophisticated machines that can handle a wide variety of substrates.^169^ Manufacturing on-site could help lower total manufacturing and operating expenses related to distribution shackles, repair, and transit. Multiple-nozzle printers are used to print big structures with the goal to suit requirements in industry. Furthermore, in an effort to standardize the manufacturing process, researchers are focusing on creating intuitive applications which can interact with any type of 3D printer.^170^ Taking into account the above-described factors, it is expected that 3D printing will soon be prepared for large-scale use, primarily for producing parts that might be very costly. Sequential manufacturing and 3D-printed facilities would be a first stage in the process of production for full adaptations; nonetheless, their ability to succeed will be largely determined by the financial implications and intricacies of the procedure.

And last, accessibility and economic viability are significant obstacles. Acceptance may be hampered by the high expense of developing new materials, the need for specialist tools, and strict legal restrictions. To close the hole and increase the commercial accessibility of such devices, cooperation between academia, business, and regulatory agencies is essential.^171^ While 3D printing holds great potential to transform the energy industry, a secure and environmentally friendly implementation will require addressing sustainability issues, ensuring thorough safety assessments, creating expandable fabrication techniques, and encouraging partnerships to increase their economic viability. Reaching the maximum potential in developing sectors will be made possible by eliminating these obstacles.

## 6 Challenges and Opportunities

Conducting polymers are often difficult in printing fine resolution structures due to the rheological properties that affects flow adhesion. Optimization of advanced printing techniques like electrohydrodynamic printing and two photon polymerization can be studied that allows for precise deposition suitable for complex structures. CPs also have significant processability restrictions and limited conductivity retention after printing. Consequently, incorporation of antioxidant stabilizers and nano additives can enhance their processability while maintaining their exceptional conductivity. Post processing techniques like UV curing can improve these factors by controlling polymers arrangement. It might be challenging to guarantee uniform conductivity throughout a 3D-printed component. The primary discussion topics in additive manufacturing focuses on increasing the uniform distribution and durability of the CPs to enable smoother printing and improved conductivity. Many CPs also lack the optimum mechanical strength and flexibility required in for structural applications. Mixing conductive polymers with mechanically strong polymeric components can enhance structural stability. These hybrid composites retain conductivity while providing the necessary support for structural integrity. However, large additions of filler materials frequently result in a brittle environment. Chemical or physical cross-linking can reinforce conductive polymers which can significantly improve the polymer’s stiffness, making it more suitable for load-bearing applications. This makes the compromise between the manufactured materials’ physical properties and scalability limit for large scale production.

In this sense, the 3D printing of CPs is a field that has just recently begun to be investigated yet has many potential uses down the road. PEDOT is the most researched CP utilized in 3D printing. Various 3D printing processes are currently being used to produce PEDOT composite-based inks. It can be treated with hydrogels, conducting additives, solvents, and other polymers to create custom inks with adjustable qualities that can be used in various applications. Tailored polymers have been synthesized with PPy and PANi, significantly increasing customization. 3D scaffolds have been made possible by employing tetramers or block copolymers. P3HT is among the many widely used conducting polymers in solar cell applications. It has not been extensively studied in the additive manufacturing artificial generators and transistors. We believe there will be plenty of potential for using this shortly.

Furthermore, we think that not enough research has been done on nanocomposites made with additive fillers and conducting polymers fabricated via 3D printing. Nevertheless, combining other bidimensional components (such as MOFs like MXene and graphene) with conducting polymers presents a significant challenge. Still, it would result in hybrid composites with various potential uses and novel applications. Furthermore, several cutting-edge printing techniques, like light-based printing, are still largely untested for conducting polymers, which could lead to new opportunities and uses down the road. Lastly, it is important to understand that ionically active gels can be manufactured using these printing methods and used for the mentioned applications. We propose that future research should focus especially on the incorporation of additional polymer since it has demonstrated potential for obtaining the required properties and reasonable conductivity. A significant rise in electrical conductivities has been obtained by 3D printing of conducting polymers. With time, they can coexist with metallic parts. We believe that substantial enhancements in the physical and electrical characteristics can be expected by preparing new equipment for additive manufacturing methods.

## 7 Conclusion

AM has undergone a rapid transformation driven by significant investments and global research initiatives. This shift has led to the widespread adoption of 3D printing across various industries, revolutionizing traditional manufacturing paradigms. One key breakthrough in AM has been the ability to fabricate highly intricate, compact, and electrically conductive designs, enabling the creation of multifunctional components. This advancement has opened up new possibilities for innovative applications in bioelectronics, stretchable and flexible electronics, sensors, and electrochemical/energy storage devices. This perspective provides a comprehensive overview of recent developments in 3D printed conductive polymers, delving into their fundamental properties and exploring their potential applications in sustainable energy devices for biomedical areas. However, despite their huge promise, 3D-printed conductive polymers face challenges in material development, printing techniques, and cost. Overcoming these will pave the way for widespread adoption and revolutionize various industries, particularly the biomedical sector.
